# Ras Activity Tunes the Period and Modulates the Entrainment of the Suprachiasmatic Clock

**DOI:** 10.3389/fneur.2017.00264

**Published:** 2017-06-09

**Authors:** Tsvetan Serchov, Rolf Heumann

**Affiliations:** ^1^Department of Psychiatry and Psychotherapy, Medical Center – University of Freiburg, Faculty of Medicine, University of Freiburg, Germany; ^2^Biochemistry II, Molecular Neurobiochemistry, Faculty of Chemistry and Biochemistry, Ruhr University Bochum, Bochum, Germany

**Keywords:** Ras, circadian, glycogen synthase kinase-3 beta, synRas mice, ERK1/2

## Abstract

The small GTPase Ras is a universal eukaryotic cytoplasmic membrane-anchored protein, which regulates diverse downstream signal transduction pathways that play an important role in the proper functioning of neurons. Ras activity is a central regulator of structural and functional synaptic plasticity in the adult nervous system, where it channels neuronal responses to various extracellular cues allowing the organism to adapt to complex environmental stimuli. The suprachiasmatic nucleus (SCN) is the principle pacemaker of the circadian clock, and the circadian and photic regulation of Ras activity in the SCN is an important modulator of the clockwork. We have generated transgenic mouse expressing constitutively active V12-H-Ras selectively in neurons *via* a synapsin I promoter (synRas mice), which serves as a suitable model to study the role of neuronal Ras signaling. Modulation of Ras activity affects ERK1,2/CREB signaling and glycogen synthase kinase-3 beta expression in the SCN, which in turn modify the photoentrainment of the clock and the fine tuning the circadian period length. The main focus of this review is to offer an overview of the function of Ras signaling in the circadian rhythm and its potential role in learning and memory consolidation.

## Introduction

Most organisms living on earth exhibit circadian rhythm controlled by autonomous timekeeping circadian clock. The circadian oscillation of intracellular clock is driven by transcription/translation-based feedback/feedforward loops, composed of a set of clock genes, as well as kinases and phosphatases that regulate the localization and stability of the clock gene protein products. Positive regulatory elements are BMAL1 and CLOCK, which form heterodimer and regulate the rhythmic transcription of Period (Per1 and Per2) and Cryptochrome (Cry1 and Cry2) genes. The PER and CRY proteins interact and translocate to the nucleus, where they act as negative regulators inhibiting further transcriptional activation. In addition to the transcriptional regulation, posttranslational mechanisms, such as phosphorylation of core clock proteins, play an important role in the regulation of the circadian clock. The casein kinases and glycogen synthase kinase-3 beta (GSK3β) have a critical function in the control of circadian period length by phosphorylating several core clock proteins, regulating their degradation, protein stability, and nuclear translocation (1–5).

In mammals, the circadian master pacemaker is located in the suprachiasmatic nucleus (SCN) of the ventral hypothalamus (6, 7). The SCN synchronize numerous biochemical, physiological, and behavioral processes in the peripheral organs with an approximate 24 h periodicity. An important feature of circadian clockwork is the ability to be reset by light, thus, allowing animals to adjust their biological rhythms to changes in the length of daytime and nighttime (6).

Recently, we demonstrated that the circadian and photic activation of the small GTPase Ras is an important modulator of the clockwork in the SCN. Ras activity fine tunes the period length and modulates photoentrainment of the circadian clock (8). The main focus of this review is to offer an overview of the function of Ras signaling in the circadian rhythm and its potential role in learning and memory consolidation.

## Ras Signaling and synRas Mouse Model

Ras is a universal eukaryotic intracellular membrane-anchored protein, which cycles between inactive GDP-bound and the signaling competent GTP-bound conformation. Several extracellular signals from multiple receptor types and intracellular second messengers converge onto the activation of Ras, including neurotrophin tyrosine kinase receptors, G-protein-coupled receptors, and local increase of intracellular Ca^2+^ concentration or Ca-calmodulin kinase II, resulting in the activation of NMDA receptors or voltage-gated Ca^2+^ channels (9, 10). Ras once activated transduces signals to several signaling pathways, including the major mitogen-activated protein kinase (MAPK)/extracellular-regulated kinase (ERK) cascade and phosphatidylinositol-3 kinase/Akt pathway. Ras plays a central role as a regulator of structural and functional synaptic plasticity in the adult mammalian brain modulating neuronal architecture and synaptic connectivity and tuning synaptic efficacy (11–14).

In order to study the role of Ras and its specific downstream effectors, we have generated a transgenic mouse model, which expresses constitutively active V12-H-Ras selectively in neurons *via* synapsin I promoter (synRas mice) (15). The synRas mice have brain hypertrophy, which results from an increased cell size and changed morphology of the pyramidal neurons (14, 15). The constitutively activated Ras increases the dendritic length, complexity, and spine density leading to a change in synaptic connectivity in the synRas mice cortex (12–14, 16). The investigation of the signal transduction in the synRas neurons showed that the expression of the constitutively activated V12-H-Ras leads to drastic increase of Ras activity and corresponding elevation of the phosphorylation level of MAPK (ERK1,2) in the cortex and hippocampus. No such changes have been observed in PI(3)K/Akt activity in adult synRas mice (15). In addition, we found increased total expression level of GSK3β (17), which might be result of enhanced Ras–MAPK signaling and ETS-p300 transcriptional complex activation (18). Furthermore, specific increases of pCREB and brain-derived neurotrophic factor (BDNF) levels in the cortex of synRas mice during the developmental stages—postnatal day 7—have been described (19).

## Ras Signaling and Photoentrainment of the Circadian Clock in SCN

**Figure 1:**
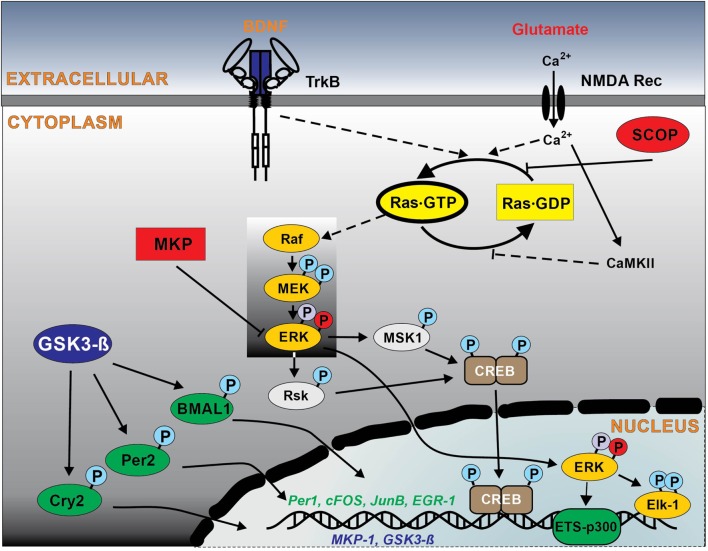
Schematic outline of intracellular Ras signaling pathways in the suprachiasmatic nucleus (SCN) regulating circadian clockwork. Solid lines show the signal pathways observed in the SCN, and broken lines indicate hypothetical pathways observed in other neuronal systems. Glutamate/NMDA and brain-derived neurotrophic factor (BDNF)/TrkB are the major ligand–receptor systems within SCN involved in the light-induced phase shifting circadian clock. The light stimuli at night induce glutamate and BDNF release, which result in activation of NMDA receptors (with a subsequent influx of Ca^2+^, activating the Ca^2+^-calmodulin kinase II) and TrkB receptor that in turn stimulates Ras. Ras is also negatively regulated by the circadian protein SCN circadian oscillatory protein (SCOP). Ras activates ERK1,2 pathway, which couples to transcriptional factors CREB and Elk-1 phosphorylation, that regulate the transcription of the immediate early genes sFos, JunB, and EGR1, clock protein Per1, the regulator of ERK1,2 pathway MAPK phosphatase 1 (MKP-1). Enhanced Ras signaling *via* ERK1,2 also activates ETS-p300 transcriptional complex, which in turn regulates circadian clock proteins modulator glycogen synthase kinase-3 beta (GSK-3β). Other abbreviations are explained in manuscript. Please note: Ras downstream effector pathways other than RAF kinase, such as PI3 kinase and Ral/GDF have been omitted for reasons of simplicity and lack of specific information in the SCN.

The potential involvement of Ras signaling in the regulation of circadian clock has been proposed in numerous studies (8, 20–27). The small GTPase Ras appears to be the major effector of BDNF-mediated signaling and one of the main upstream regulators of ERK pathway resulting in elevated levels of CREB phosphorylation (19) (Figure 1). Indeed, the activation of MAPK pathway and particularly ERK1,2 and its coupling to the activation of transcription factors Elk-1 and CREB (28, 29) is an important molecular mechanism for photoentrainment of the SCN (Figure 1). *In vivo* studies have shown that inhibition of ERK1,2 in mouse SCN attenuates both the phase shifting effects of light (28, 30) and immediate early gene expression (31). BDNF and its receptor, TrkB, are also necessary for photic resetting. BDNF protein levels oscillate in the SCN with high levels at night, when photic stimulation and glutamate can reset the circadian clockwork (32). The inhibition of TrkB receptors blocks photic- and glutamate-induced clock resetting (33, 34).

Consistently, photic stimulation at early and late subjective night activates Ras in the SCN (8) and Ras activation correlates with the length of the light exposure (20), suggesting a direct involvement of Ras in the signaling pathways, coupling photic input to the SCN clock. The light stimuli induce glutamate release from the nerve terminals of the retino-hypothalamic tract, which results in activation of NMDA receptors with a subsequent influx of Ca^2+^ (35, 36), activating the Ca^2+^-calmodulin kinase II that in turn stimulates Ras (9, 10) (Figure 1).

Direct evidences for the involvement of Ras in the molecular mechanisms that adjust the circadian clock to the light/dark cycle come from the synRas mice (8, 25). The enhanced Ras activation in the SCN of synRas mice leads to potentiation of the light-induced phase delays at early night and total inhibition of the light-induced phase advances at late night of spontaneous locomotor activity (8). The magnitude of Ras-regulated ERK phosphorylation correlates with the extent of the phase delays at early subjective night—with stronger ERK activation leading to larger phase delays in circadian behavior (20, 37). ERK1,2 phosphorylates p90 ribosomal S6 kinase, which in turn phosphorylates CREB, required for the photic resetting of the SCN (38, 39). In addition, ERK1,2/CREB pathway couples light to immediate early genes expression c-Fos, a robust marker of SCN activation by photic stimuli, and the induction of the clock gene PER1 (31, 40, 41). Therefore, the enhanced activation of the Ras/ERK1,2/CREB pathway in the SCN of synRas mice at early subjective night leads to increased phase delays and enhanced photic induction of c-Fos protein expression in the SCN. Though several reports have shown that the photic stimulation of ERK1,2/CREB phosphorylation is an essential event for the clock photoentrainment, the activation of this pathway is not sufficient to induce c-Fos expression and phase advance the clock of synRas mice at late subjective night (8, 37). Thus, the enhanced basal levels of activation of Ras/ERK in the SCN of synRas mice at early subjective night phase delay the circadian clock and compensate the photic-induced resetting in the late subjective night.

## Ras Signaling and Circadian Period Length

Several reports demonstrated circadian oscillation of Ras activity in various brain regions and peripheral organs, including chick pineal gland (23), mouse hippocampus (22), liver (26), and SCN (8). Potential regulator of Ras activity in the brain is the SCN circadian oscillatory protein (SCOP) (42). The expression of SCOP reach peak levels during late subjective night to inhibit the Ras/ERK pathway by binding to the nucleotide-free state of Ras and preventing the binding of GTP (42, 43) (Figure 1). Furthermore, Ras is one of the main targets for neurotrophins (44). BDNF mRNA and protein levels show a circadian oscillation in different regions of the brain (32, 33, 45–48). Given that multiple extracellular signals such as growth factors and cytokines can stimulate Ras activation in a context-dependent manner, circadian oscillations of circulating humoral factors may lead to rhythmic Ras activation in the liver.

Numerous studies support a model that the circadian activation of ERK1,2 is regulated by the oscillating activation of Ras *via* the classical Ras–MAPK pathway, which is commonly involved in numerous intracellular events. The Ras-mediated regulation of ERK1,2 activity is conserved mechanism used in many clock-containing tissues, such as the mammalian SCN, as well as in other regions, like hippocampus, pineal gland, and liver (8, 22, 23, 26, 30). Indeed, the data from synRas mice show that the enhanced Ras activity increases ERK1,2 phosphorylation at early subjective night (8). However, the circadian regulation of pERK1,2 levels was preserved, as result of the rhythmic oscillation of the endogenous Ras and the rhythmic expression of MAPK phosphatase 1 (MKP-1) (8, 20) (Figure 1). MKP-1 shows circadian oscillation with the peak time at night in mouse liver and the SCN (8, 26).

It has been recently shown that modulation of Ras activity affects the period length (τ) of circadian oscillation (8). Enhanced activation of Ras in the SCN of synRas mice results of shortening of τ, while *in vitro* inhibition of Ras activity lengthens the circadian oscillation of BMAL1 promoter-driven luciferase activity (8). By contrast, inducible overexpression of Ras in cancer cell lines disrupts the circadian clock enhances the circadian period, while Ras inhibition leads to a shortening of period length, as mathematically predicted by simulations of BMAL1-mediated transcription (24). However, the mechanism of Ras-mediated modulation of the circadian period length is not well investigated yet. Though, the fine tuning of the molecular clock might have a tumor suppressive role in Ras-driven lung cancer (49).

Within the regulation of τ, phosphorylation of core clock proteins plays an important role, as it determines their stability and degradation (1–3). GSK3β acts as one of the upstream kinases phosphorylating several clock proteins, such as CLOCK, BMAL1, PER 2, Rev-erbα, and CRY2 (1, 4, 5, 50–53) (Figure 1). Enhanced Ras activity results in high total protein expression and low levels of deactivating phosphorylation of GSK3β in the SCN (8), as well as increased GSK3β activity in the cortex of synRas mice (17). Moreover, the inhibition of Ras activity decreases protein expression and increases inhibitory phosphorylation level of GSK3β *in vitro* (8). Thus, the increased GSK3β activity leads to a shortening, whereas a decreased function leads to substantial lengthening, of the circadian period length (1, 51, 52). By contrast, other studies show that GSK3β inhibition shortens the circadian period *in vitro* (50), while its chronic activation lengthens the period of mice (54). Interestingly, Ras-mediated regulation of τ may also be a result of the direct influence of Ras on the MAPK pathway. Reduction of MAPK signaling by deletion of MSK1, a target kinase of ERK1,2, results in a lengthened period of circadian behavior (55). In addition, downregulation of ERK1,2 activity inhibits the rhythm and dampens the basal level of the expression of several clock genes (56). Taken together, these data suggest that changes in GTP-Ras levels influence τ *via* modulation of ERK1,2 and GSK3β activity.

## Role of Ras in Learning and Memory Consolidation

The circadian clocks regulate various neural functions, including cognitive performance. Several studies have demonstrated diurnal modulation of learning and memory in different paradigms, such as Morris water maze task (57), novel object recognition task (58), and fear-related tasks (59). Many investigators have linked Ras and ERK1/2 to learning and memory, since temporal modulation of ERK1/2 activation by Ras is known to play a critical role in several forms of neuroplasticity (60). Spatial and declarative memories are processed in the hippocampus (61), where Ras/MAPK pathway and the downstream CREB transcriptional pathway play an important role. Indeed, it is reported that Ras, ERK, and CREB activities show daily (basal) fluctuations in the mouse hippocampus (22). It has been recently shown that the consolidation of long-term recognition memory is a circadian-regulated process, mediated by the Ras-inhibitory protein SCOP (62). On the other hand, synRas mice showed impaired spatial short-term memory associated also with a reduced proliferation of newborn cells in the *dentate gyrus* of the hippocampus (63, 64) and decreased short-term recognition memory (65, 66). All these studies suggest that modulation of Ras activity is critical for memory performance, but a question remains as to whether and how circadian regulation of Ras is associated in this process.

Several reports suggest that a disordered circadian system is implicated in the etiology and symptomatology of many psychiatric disorders. Interestingly, the therapeutic action of lithium, an effective mood stabilizer for bipolar affective disorder, may be related to direct effects on the circadian clock *via* the inhibition of GSK-3β (51, 67). Although this enzyme has a number of functions that could potentially mediate the therapeutic effects of lithium (68), one possibility is *via* its function as a central regulator of the circadian clock. Consistently, it has been shown recently that activation of GSK-3β may link to the activity in the SCN neurons by regulating their persistent sodium currents (69). However, in order to understand how timing of action potentials is coupled to the pacemaker activity in the SCN, it still needs to be investigated how Ras signaling encodes electrical activity specifically in the SCN neurons.

## Conclusion

**Figure 2:**
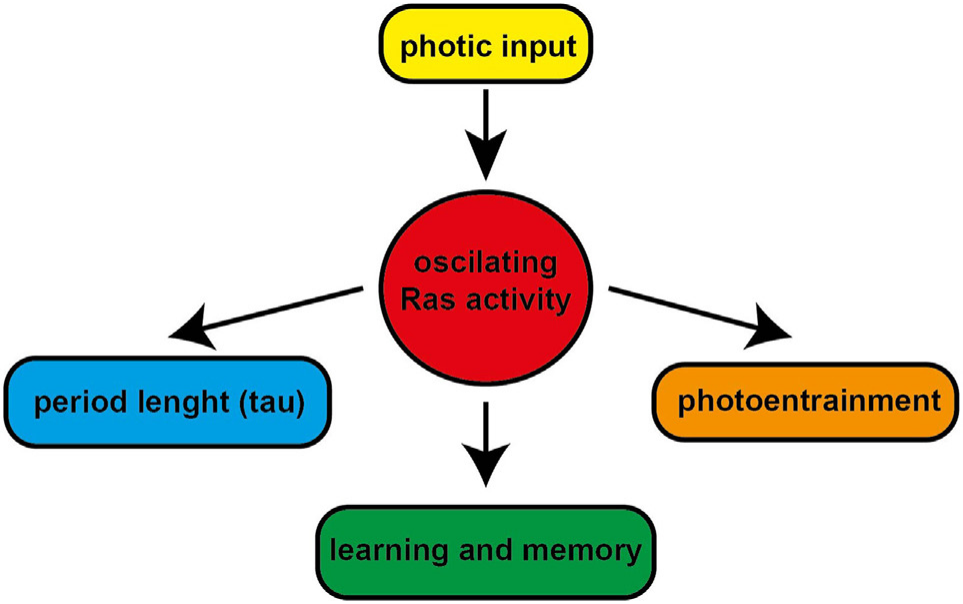
The role of Ras in the regulation of circadian clockwork and learning and memory. The circadian and photic regulation of Ras activity in the suprachiasmatic nucleus modulates the light-induced phase resetting of the clock and fine tunes the circadian period length. The circadian modulation of Ras signaling might have potential role in learning and memory.

The small GTPase Ras activity plays a role as a central regulator of structural and functional synaptic plasticity in the nervous system, where it mediates neuronal responses to various extracellular cues allowing the organism to adapt to complex environmental stimuli. Thus, the circadian and photic regulation of Ras activity in the SCN is an important modulator of the clockwork influencing the light-induced phase resetting of the clock and fine tuning the circadian period length. Furthermore, the circadian modulation of Ras signaling might have potential role in memory consolidation and mood regulation (Figure 2). However, the involvement of Ras-controlled GSK-3β expression and its mechanism of regulation in the pathophysiology of bipolar disorder still remain to be investigated.

## Author Contributions

TS wrote the manuscript with assistance from RH.

## Conflict of Interest Statement

The authors declare that the research was conducted in the absence of any commercial or financial relationships that could be construed as a potential conflict of interest.
